# Sensor Fusion for Social Navigation on a Mobile Robot Based on Fast Marching Square and Gaussian Mixture Model

**DOI:** 10.3390/s22228728

**Published:** 2022-11-11

**Authors:** Alicia Mora, Adrian Prados, Alberto Mendez, Ramon Barber, Santiago Garrido

**Affiliations:** Robotics Lab, Universidad Carlos III de Madrid, 28911 Madrid, Spain

**Keywords:** sensor fusion, social navigation, multi-layer map, fast marching square, mixture Gaussian model

## Abstract

Mobile robot navigation has been studied for a long time, and it is nowadays widely used in multiple applications. However, it is traditionally focused on two-dimensional geometric characteristics of the environments. There are situations in which robots need to share space with people, so additional aspects, such as social distancing, need to be considered. In this work, an approach for social navigation is presented. A multi-layer model of the environment containing geometric and topological characteristics is built based on the fusion of multiple sensor information. This is later used for navigating the environment considering social distancing from individuals and groups of people. The main novelty is combining fast marching square for path planning and navigation with Gaussian models to represent people. This combination allows to create a continuous representation of the environment from which smooth paths can be extracted and modified according to dynamically captured data. Results prove the practical application of the method on an assistive robot for navigating indoor scenarios, including a behavior for crossing narrow passages. People are efficiently detected and modeled to assure their comfort when robots are around.

## 1. Introduction

Nowadays, there is a rising popularity of mobile robots for multiple applications, both outdoors and indoors. Advances in technology have turned robots into a powerful tool for multiple applications, such as preventing people from performing demanding and repetitive tasks or assisting them when their capacities have deteriorated. The work performed to date in this field has reached a point where robust algorithms for mobile robot navigation are available, especially when talking about 2D techniques. Robots can map environments with algorithms such as SLAM and then localize themselves in the resulting map to plan a trajectory using a single sensor, typically a 2D LiDAR or a depth camera. However, these tasks are purely based on geometric information. Even though they can be adapted to dynamic environments where moving obstacles need to be avoided, a meaning is not assigned to any of these events. Situations in which robots and people need to coexist are highly acclaimed in locations such as museums, hospitals or residences for elderly people. For these cases, a differentiation is required between people and any other object because social conventions must be kept. In particular, in the case in which robots need to interact with people, their social distance must be respected. This is known as social navigation. In contrast to traditional navigation, the aim is to reach a goal while following social conventions [1]. One of the main challenges mentioned on the cited work is modeling social space or proxemics, defined as the space around a person that, if intruded upon, could cause discomfort.

In this paper, we propose a multi-sensor approach for solving the traditional robot navigation problem with the advantage of detecting and modeling people. Initially, the environment is mapped using 3D information, which broadens the data-capturing range with respect to traditional 2D techniques. Then, a topological map is extracted to locate rooms and narrow passages, allowing the application of different navigation approaches depending on the robot location. This is especially helpful for high-dimensional robots, which need to traverse narrow passages carefully. Additionally, the resulting map is directly understandable by humans, providing robots with higher-level concepts that are closer to how we interpret our surroundings so that they become integrated in the scene [2]. Finally, people are detected, and their personal space is modeled during navigation to keep the dynamic behavior of the system. The resulting navigation strategy is applied on a practical case in which an assistive robot needs to navigate an indoor scenario. A key detail of the proposed architecture is using fast marching square for planning and navigating, which defines the geometric space in a continuous mode. This allows to apply Gaussian functions to model people since their continuous space can be fully considered and easily integrated.

State-of-the-art works have already approached the social navigation problem. One of the main research lines on the field is based on social force models (SFM). Similar to potential field approaches, this method models people as a repulsive force, whereas the goal is considered an attractive force. The main issue of this method is that peoples’ discomfort is not considered because their motion is not taken into account. This is corrected in some approaches, including techniques such as defining motion primitives [3] or predicting their trajectories [4].

Another well-known approach is modeling the social distance using Gaussians. In [5], a similar approach to ours is proposed in order to consider socially acceptable behaviors when the robot navigates. People are clustered and modeled using a Gaussian mixture. However, the Gaussian is simplified by a polygonal chain, losing its continuous values. Additionally, only discrete planners are applied, which could derive into uneven paths or the requirement of an extra step to smooth them. People found on a scenario are also defined by Gaussians in [6]. In this case, only the closest person is considered and modeled. Hence, no distinction is performed between individuals and groups. The same authors in [7] grouped people using Delaunay triangulation and modeled them using asymmetric Gaussians. Again, as it happened in previously cited works, the Gaussian is discretized with level curves in both works so that the initial path, calculated with A*, is modified to be inside a certain region. Authors in [8] proposed the use of human emotions to determine how close the robot can navigate from people. Although this is a promising approach, the provided results are only virtual. No specifications about how to determine peoples’ emotions are provided, especially when their facial expressions cannot be observed (i.e., when a person is standing with their back to the robot). Moreover, the path planning strategy is discrete, so the resulting paths are not smooth.

Other techniques include the usage of external devices placed on individuals. Users in [9] are provided with a wearable haptic interface which vibrates depending on the robot intention. These methods are highly dependent on the device range and are limited to the number of people wearing it, so they are not generalizable to crowded situations.

In contrast, our approach provides the following contributions:

- Multiple sensors are fused for mapping and socially navigating scenarios: a 2D LiDAR, a 3D LiDAR and an RGBD camera.
- Three-dimensional information is captured to extract a geometric and topological map, which will serve as the basis for path planning and navigation.
- A navigation strategy using the fast marching square method is designed, considering static and dynamic objects.
- A behavior for narrow passage trespassing is designed to avoid collisions in robots with high dimensionality.
- People are detected and modeled using Gaussian functions considering social distance. A differentiation is made between individuals and groups. Additionally, the Gaussian model is fully considered, with no discretization step required. The use of fast marching square facilitates the addition of Gaussians into the model.
- The method is tested on a real scenario in which a domestic robot coexists with people.

In the following sections, the proposed architecture will be explained in more detail.

## 2. Materials and Methods

### 2.1. Robotic Platform

The robotic platform used in this work is a mobile bimanipulator robot named ADAM. Its design is thought for assisting elderly people in tasks that require manipulation, mostly cooking. It is part of the HEROITEA project (Heterogeneous Social-Mobile Manipulator Robot Intelligent Teams for Elderly-People Assistance). Apart from manipulating capabilities, it is essential for the robot to navigate the environment, being conscious of where people are. That is why it was chosen as the experimental platform for the proposed social navigation strategy.

**Figure 1:**
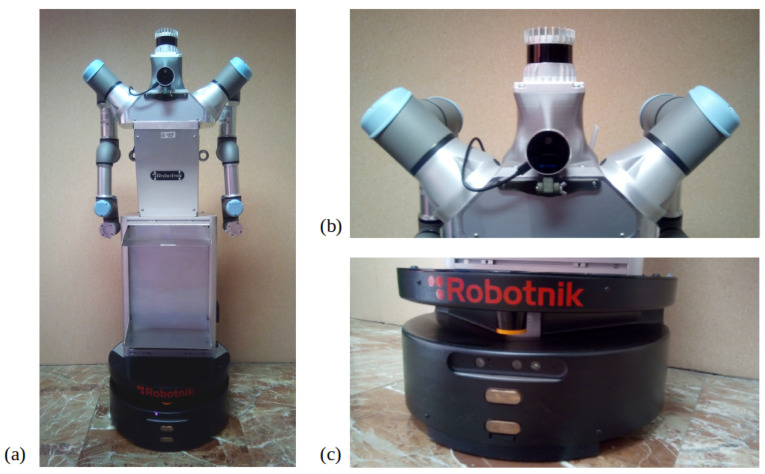
ADAM robot: (**a**) general overview of the mobile robot, where the robotic base, the torso and the two arms can be observed, (**b**) 3D LiDAR and RGBD camera placed at the top of the torso, (**c**) 2D LiDAR placed on the robotic base.

ADAM is formed by an omnidirectional mobile base, a torso and two 6 DoF industrial arms, as shown in Figure 1. Its total height is 1.6 m, and its base has a 0.6 m diameter. This means that the robot has a similar height with respect to a person, but its width is bigger, especially when the robotic arms are not very close to the torso. This fact needs to be taken into account when navigating narrow zones, which mostly correspond to doors in indoor scenarios.

With respect to sensors, ADAM is a multi-sensor robot. For this application, a total of four sensors are used, one of them being propioceptive and the other three, exteroceptive. The first one is the set of encoders placed on the robotic base wheels. They will serve for calculating the odometry, which is required in the mapping and localization modules. Regarding exteroceptive sensors, the three selected ones are a 2D LiDAR, a 3D LiDAR and an RGBD camera. The 2D LiDAR, a Hokuyo UST-20LX [10], is integrated in the robotic base, slightly displaced above ground. Due to its position, large objects, such as tables, cannot be fully detected. That is why its main objective is detecting objects on a low-level basis during navigation. It has a total of 987 light rays, which cover 270° and a mid-detection range of 10 m/20 m. The 3D LiDAR, an Ouster OS0 [11], is placed at the top of the torso, so it is capable of measuring objects at multiple heights for mapping. It has a total of 128 channels with a 90° field of view and a 35 m range at 10%. Finally, the RGBD camera is a RealSense L151 [12], consisting on a 2MP RGB sensor with a resolution of 1920 × 1080 px and a depth sensor with LiDAR technology with a range from 0.25 m to 9 m and 70° × 55° (±3°) field of view. It is placed below the 3D LiDAR on the front part of the robot. Its main objective is detecting people when the robot is navigating so that it does not traverse their personal space.

Given these characteristics, each sensor has a different application. The 3D LiDAR has a wide range for capturing data since it captures information at 360° horizontally and 90° vertically with a mid-detection range of 10 m/20 m. Hence, it is used for mapping the environment. The mapping procedure is sped up because a wide range of data is available with small robot displacements. The 3D camera could be also used for this task, but its field of view and range are highly reduced with respect to the Ouster sensor, so more time would be required for mapping, and some data would still be missing. That is why its functionality is applied for people detection while navigating, fusing RGB and depth information. The 2D LiDAR complements the camera information because it has a wider detection angle and range, so a higher number of collision threats can be detected. The 3D LiDAR is not used during navigation because of its high data quantity, which, combined with the people detection module, could reduce the system time response, so the task may not be achieved in real time.

**Figure 2:**
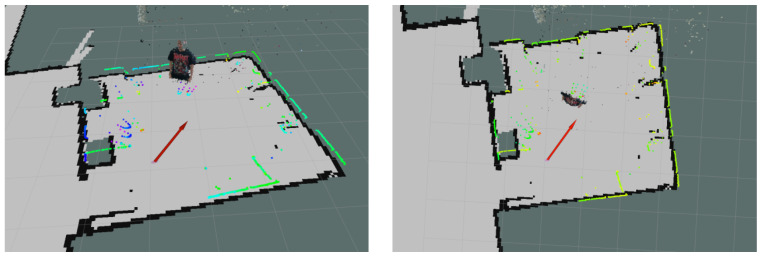
Side view (**left**) and top view (**right**) of aligned data from multiple sensors. Greenish dots correspond to the 2D LiDAR, and the colored point cloud corresponds to the RGBD camera. The robot pose is represented with the red arrow. The two U-shaped elements captured with the 2D LiDAR at the center of the room (person feet) are correctly aligned with the person captured in the point cloud.

Regarding software, the selected platform for exchanging information among sensors, actuators and the mapping and navigation algorithms is ROS. The robot is made of elements from diverse companies, so ROS allows to communicate every part with no synchronization issues. Moreover, additional elements can be easily integrated in the robotic system. This is the case of the 3D LiDAR and the RGBD camera. The mobile robot only counts with a 2D LiDAR on its base, so the other two sensors were mounted on the robot using customized 3D-printed pieces. When performing this task, it is essential to correctly define the frame tree so all sensors are referenced with respect to the same reference frame. Figure 2 shows an example of aligned data from the 2D LiDAR and the RGBD camera, proving that the frame tree is properly configured. Mapping and navigation algorithms are programmed in MATLAB, which can be easily connected to ROS using the ROS toolbox.

### 2.2. Navigation System

Robot navigation has been defined by multiple authors in the literature. The authors in [13] define mobile robot navigation as the capability of passing a test in which the robot is placed in an unknown environment that it has to explore and then go to a specified place, minimizing a cost function such as time or distance. In [14], the problem of achieving an autonomous robot navigation is divided into four subtasks: identifying the current robot location, determining a path to the objective, avoiding collisions, and resolving any conflicts between the previous two subtasks. More recently, works such as [15] are starting to include higher-level information in the robotic navigation systems. In order to achieve a better autonomy level in robots, it is not enough to only indicate a geometric target that the robot has to reach. New trends propose to create navigation systems, taking into account how humans interpret their surrondings, so in addition to the classical notion of autonomous navigation, the system needs to be designed in a human-friendly manner. According to these definitions, the proposed navigation system is formed by four modules:

- Mapping: the robot needs to construct an environment representation based on sensor information. For this application, a multi-map system is proposed. It is formed by two layers, geometric and topological.
- Localization: a precise localization is required to know where the robot is placed. In this work, it is applied on the geometric map level.
- Path planning: since this module is highly influenced by the selected mapping procedure, it is based on a geometric and topological level as well. The planner finds a global topological plan and local geometric paths.
- Plan execution: once the three above-mentioned modules are available, the robot needs to follow the calculated instructions. Hence, this module is performed in real time. This implies taking into account unknown static and dynamic objects that interfere with previous knowledge. Additionally, given the high dimensions of the robotic platform, specific behaviors need to be defined to avoid collisions in narrow zones. Finally, this module includes the social navigation perspective by detecting people and modeling their personal space.

These modules are explained in a more detail in the following subsections.

#### 2.2.1. Mapping Based on 3D Information

The most common way of representing the environment for mobile robot navigation tasks is using geometric information. Traditionally, 2D SLAM techniques are applied. However, these methods are very limited to the height in which the 2D LiDAR sensor is placed. Given that it is normally positioned at the robot base, measurements are captured at a height slightly displaced above ground, so obstacles such as tables or chairs are not fully captured. Hence, there are obstacles that are not being captured but which represent a collision threat. In this work, it is proposed to use a 3D LiDAR for obtaining information at multiple heights. The main objective is finding a 3D representation of the environment from which 2D occupancy grid maps can be created by taking 3D information slices. The selected algorithm for creating the 3D point cloud representation is SLAM based on Harmony Search, as described in [16].

**Figure 3:**
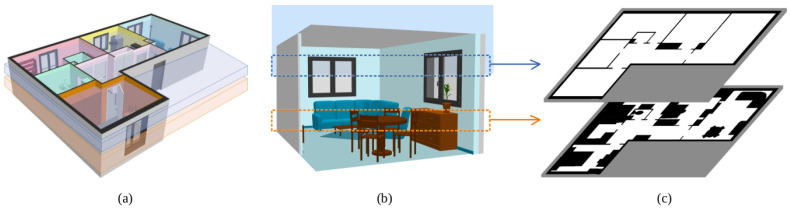
The 3D slices for 2D mapping: (**a**) initial scenario, where the slices are marked in blue (layout space) and orange (navigation space), (**b**) location of the 3D slices inside a room, where the layout space is empty and the navigation space contains furniture, and (**c**) resulting 2D occupancy grid maps.

For the proposed application of navigating indoor environments, two 2D occupancy grid maps can be useful. The first one is the traditional occupancy grid map used for navigation tasks (localization and path planning). For that reason, it is proposed to use a slice of 3D data from the robotic base to the total height of the robot, collecting every geometric information that represents a potential geometric limitation. The second one is focused on describing the layout of the indoor environment in which the robot is placed. The aim is finding a geometric segmentation of the environment based on narrow passages, mainly doors. Given that removing objects such as furniture significantly improves results, in this case, the 3D slice is taken above the robot’s height and below the ceiling. The result is an occupancy grid map in which the core structure of the indoor scenario is represented. This procedure is visually represented in Figure 3, where the 3D slices are marked on an indoor scenario, and the two proposed occupancy grid maps are extracted.

It has already been stated that geometric maps are useful for robots to navigate indoor scenarios. However, this representation is far from how people understand their surroundings. When we think about moving around an indoor location, we tend to first identify the different rooms and narrow passages, or doors, that we need to traverse. By analyzing the geometric properties of occupancy grid maps, these can be divided into meaningful regions. More precisely, the aim is finding narrow passages representing separations. In this way, robots are brought closer to our way of thinking. Additionally, tasks such as path planning are sped up when combining a topological global planner with a geometric local planner, as proved by many works, such as [17,18]. The selected method for segmenting the environment is presented in [19], where Voronoi diagrams are extracted from both free and occupied regions in an occupancy grid map to determine region separations. The provided results prove the effectiveness of the method. The outcome is a labeled map, which will be used for extracting the final topological map.

**Figure 4:**
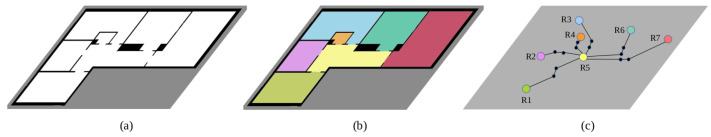
Topological map extraction: (**a**) initial occupancy grid map, (**b**) segmented map, where each color corresponds to a different location, (**c**) topological map, where colored nodes correspond to rooms and black dots indicate door nodes.

The proposed topological map is formed by nodes representing indoor locations, mainly rooms, corridors and narrow passages or doors, and edges representing their connectivity. Initially, nodes are defined for both rooms and doors. With respect to rooms, a node is assigned to every labeled region, additionally saving their perimeter values. As for doors, located in label boundaries, two nodes are created, one on each side of the separation. This will be useful for traversing doors when navigating due to the large dimensions of the experimental robotic platform. One node will serve for approaching the door, and the second one will serve as the reference point for moving. This navigation strategy is explained in more detail in Section 2.2.4. In the case of door nodes, their corresponding geometric coordinates are saved. Finally, edges are created by checking with labels that coincide on each separation. Figure 4 summarizes this procedure.

#### 2.2.2. Localization

Localization is carried out in the geometric level using sensor information from the 2D LiDAR placed on the robot base. It was decided to apply the localization tools provided by ROS navigation stack [20]. The selected method is AMCL (adaptive Monte Carlo localization), a probabilistic method based on particle filter.

#### 2.2.3. Path Planning

Planning on large geometric maps is time-consuming. For this reason, it is proposed to first plan on the global topological map and then plan geometrically on each separate location. Given the coordinates in which the robot is placed (provided by the localization algorithm) and target point, room perimeters are checked to see if they contain the points. The node containing the localization point will be the starting node, and the one containing the target point will be the end node for the topological planner. Then, Dijkstra [21] is applied to obtain the sequence of rooms and doors that need to be traversed. For this application, weights for Dijkstra are defined by the distance between nodes.

Then, geometric paths are locally planned for each room. This step is only focused on open areas given that doors are only separations and they will be traversed in a different manner to avoid collisions with door frames. The selected planner is FM2 (fast marching square) [22]. The original concept of Fast Marching is based on the way in which light is propagated in space, which, applied to an occupancy grid map, results in a matrix, where each cell indicates the arrival time of the wave. This matrix is called *velocity map* and it is denoted by *F*. Velocity map values range from zero to one, where zero values indicate occupied space, and one corresponds to zones of the maximum allowed velocity for the robot because they are far enough from obstacles. FM2 is capable of finding the shortest path on *F* while optimizing speed, that is to say, time. Some major highlights of this method are the capability of finding the fastest possible path, being smooth and avoiding the local minima.

**Figure 5:**
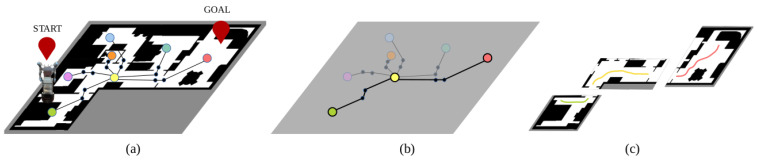
Planning steps: (**a**) initial scenario representation, where red signs indicate the starting and goal positions, (**b**) global topological plan (highlighted part of the map), and (**c**) local geometric plans. The same colors are used on the topological and geometric plans to indicate the relation among them.

Figure 5 summarizes the planning steps. Once the geometric and topological maps are available, and hence aligned, the global topological plan is calculated, as well as the geometric paths on each individual room, each one corresponding to a topological room node.

#### 2.2.4. Plan Execution

**Figure 6:**
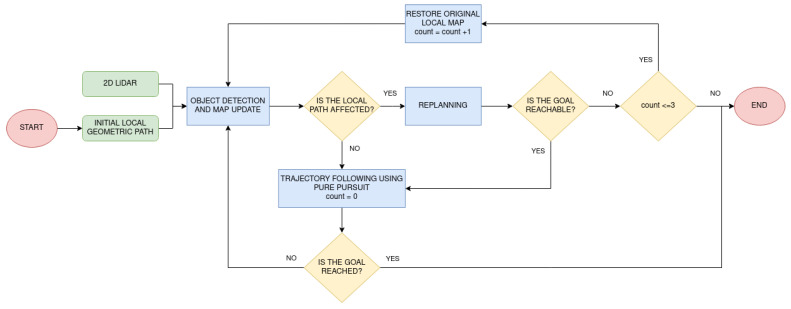
Robot plan execution including dynamically detected objects.

The third module for robot navigation is executing the planned path. It is based on the definition of two main robot behaviors, one for moving on wide areas and another one for traversing doors. In the first case, the robot follows the trajectory provided by FM2 using Pure Pursuit controller [23]. If an unknown obstacle is detected by the 2D LiDAR, it is added to the map. If it interferes with the path that is being followed, FM2 is applied again in the updated map. This method allows to avoid obstacles in real time. In the case of including dynamic obstacles in the map, such as people passing by, a situation in which the robot is trapped on the map can happen. However, it can occur that mapped people are not there anymore. When this happens, the map is returned to its original values and the path is replanned. If obstacles are found again three consecutive times and a path is not feasible, the whole process stops. This could happen, for instance, if the robot was surrounded by people for a long time. A summary of the robot behavior during plan execution is represented by the flowchart in Figure 6.

**Figure 7:**
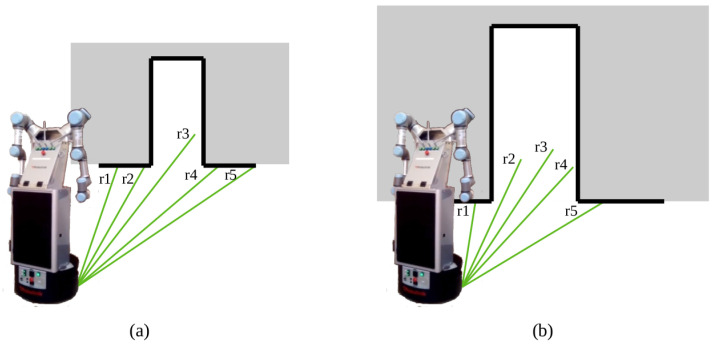
Door-crossing behavior where LiDAR rays, marked in green, are used for correcting the robot pose: (**a**) r2, r4 and r3 are checked (**b**) r1, r5 and r3 are checked.

The second robot behavior occurs when the robot needs to traverse a narrow passage. Given the large dimensions of the robotic platform, its movements need to be continuously checked to avoid collisions. For that reason, a low level functioning based on the 2D LiDAR is proposed. It counts with two steps: approximation and crossing. Initially, the robot moves to the closest door node and is rotated toward the next node, symmetrically placed at the other side of the door. Then, the crossing step is performed by assigning a constant linear velocity to the robot wheels. Due to irregularities in real scenarios, such as floor bumps, the robot tends to leave the straight line that it has to follow. To avoid lateral collisions, the 2D LiDAR measures distances to the closest obstacles on each side. More specifically, five LiDAR rays are checked on this step, as shown in Figure 7. LiDAR rays are numbered on the robot platform from left to right, starting at 1 and ending at 987. For this application, rays 293 and 393 are selected on the left side, corresponding to r1 and r2 on the drawing, and rays 593 and 693 are selected on the right side, corresponding to r4 and r5 on the drawing, respectively. r3 is placed at the central part, corresponding to ray 493. Hence, the selected rays are symmetrically placed on each of the LiDAR sides. Initially, r1, r2, r4 and r5 hit the wall surrounding the door, so rays r2 and r4 are checked. If the measured distance is smaller on one of the sides, an angular velocity is added with the opposite direction to compensate for the error. When the robot gets closer to the door, these rays stop hitting the wall, so rays r1 and r5 are used instead. These are not checked from the beginning because they are further away from the door, and other obstacles nearby could interfere in the performed comparisons because distances are affected. Finally, the distance information at the front ray r3 is also checked to see if a dynamic object, such as a person passing by, is found. In such a case, the robot stops until the space is again free.

#### 2.2.5. Modeling People for Social Navigation Strategies

**Figure 8:**
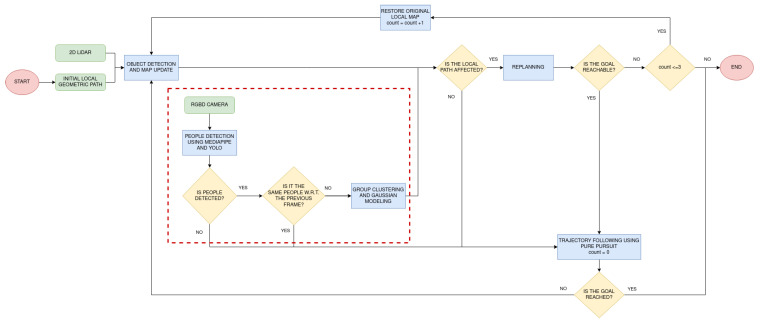
Modification of the plan following technique, marked with a red discontinuous rectangle. Camera data are used for locating people and modeling them.

Up to this point, no differentiation has been made between objects and people. When the robot detects a collision threat with the 2D LiDAR, it is automatically inserted into the occupancy grid map without checking whether it is a person or not. In this section, the social navigation module is integrated with the above explained navigation system. More specifically, people are considered during navigation on wide zones in the plan execution phase since, initially, no information about people location can be obtained. Only when the RGBD camera detects a person is the social navigation module activated. A summary of the proposed modification is shown in Figure 8. Apart from including detected objects, the camera indicates whether people are found in front of the robot. In such a case, they are modeled and considered in the path following behavior. Hence, two steps are performed in this phase: people detection and personal space modeling. An additional verification before including people is performed to avoid modeling the same person multiple times. An explanation of how these steps work can be hereunder found.

##### People Detection

**Figure 9:**
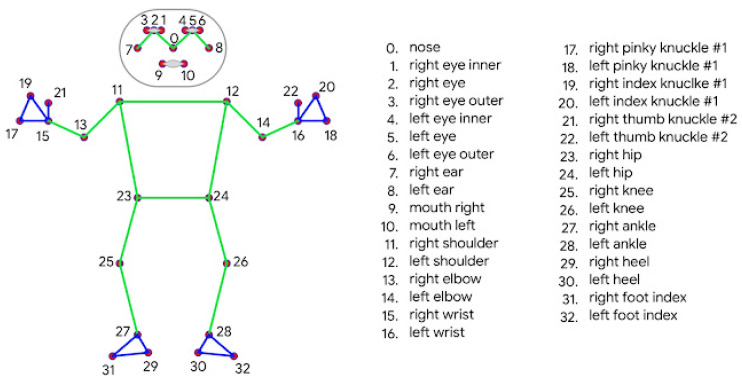
List of 33 markers used by MediaPipe and their position in the human body to estimate their pose [30].

Real-time people detection has had a great development in recent years, with diverse techniques and applications in several research fields [24,25,26]. In this paper, it was decided to combine MediaPipe [27] and YOLO [28]. MediaPipe is an open source library developed by Google for human pose estimation. It can be easily integrated with multiple platforms, such as ROS, and it can be GPU accelerated. It extends the 17 body markers from the COCO topology (green dots in Figure 9) to 33 markers defined in BlazePose, improving pose prediction. However, it can only detect one person at a time. In order to enable multi-person skeletonization, people are initially detected using YOLO. YOLO(You Only Look Once) is a real-time object detection system. The main difference with other systems is that it does not perform a search by regions in the image determining whether there are objects to be detected or not. In contrast, it is able to perform a global search within the whole image. In this paper, YOLOv5 version is chosen, given that it counts with better accuracy than its predecessors [29]. It calculates bounding boxes where people are found. Then, MediaPipe is applied on each box separately.

##### 3D Pose Estimation

In order to perform social navigation, 3D people poses are required to define their personal space. The process can be divided into three main tasks: 2D pose estimation, 3D correspondence and reference frame change.

The first task consists of detecting people and estimating their pose in the 2D plane corresponding to the RGB image. As mentioned before, YOLO finds the bounding boxes in which people are found, and MediaPipe estimates their skeleton. Only three markers are selected, and their pixel coordinates are saved—0, 11 and 12, corresponding to nose, right shoulder and left shoulder, respectively. This choice was made according to the camera location. It is placed at the height of the robot shoulders (1.3 m high approximately), so these points are better detected than, for instance, those corresponding to the hips, which could be under the image boundaries. Shoulder points are used for estimating the central point of the person with their mean value. Detecting the nose is particularly important in cases in which the person’s orientation cannot be estimated using the point cloud, as it will be later explained.

**Figure 10:**
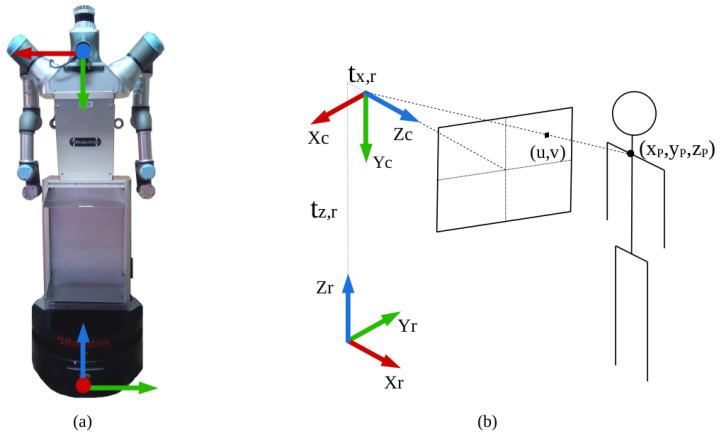
System reference frames: (**a**) camera (top) and base (bottom) frames placed on the robot, (**b**) camera frame (Xc,Yc,Zc) and base frame (Xr,Yr,Zr), where $t_{x,r}$ and $t_{z,r}$ indicate translations. The line joining the camera origin and the pixel (u,v) is defined by *projectPixelTo3dRay*, and *cKDTree* finds the 3D point (Xp,Yp,Zp).

The second task consists of turning each 2D point of interest from pixels to a 3D point in meters with respect to the camera reference frame. For this purpose, the pinhole camera model and the image point cloud are used. Given a pixel coordinate (u,v), the function projectPixelTo3dRay from the image_geometry ROS package [31] returns the 3D ray passing through (u,v), represented as a 3D vector. Then, the function *cKDTree* from the Python Spatial Algorithms and Data Structures library [32] finds the closest point on the point cloud to the estimated 3D location. The result is the desired 3D point with respect to the camera reference frame. A schematic representation of this procedure is found in Figure 10. This is applied using a region of interest around the desired pixel so that an average depth value is obtained, filtering out errors.

The third and last task is changing the 3D points from the camera reference frame to the robot base reference frame. These are visually represented in Figure 10. Two rotation transformations are required: a +90° rotation in the X axis followed by a +90° rotation in the Z axis. Finally, there is a translation in the Z axis corresponding to the difference in height between the robot base and the camera as well as a translation in the X axis because the camera is slightly in front of the center of the robot base, where the reference frame is positioned.

**Figure 11:**
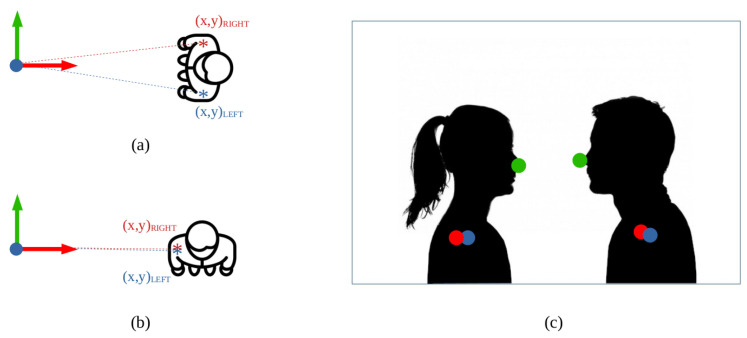
Extracting orientation from skeletonization: (**a**) if shoulders are clearly distinguishable, coordinates ${\left(x,y\right)}_{RIGHT}$ and ${\left(x,y\right)}_{LEFT}$ are checked to calculate the desired angle, (**b**) when the person is sideways, both shoulder coordinates coincide, so an extra point needs to be checked, (**c**) the RGB image is used to see where the nose (green dot) is placed with respect to the shoulders (red and blue dots), assigning a different predefined value depending on if it is to the left or to the right.

After performing the three tasks, the result is a set of 3D points corresponding to the person’s central point and the two shoulders with respect to the robot base. These data are finally used for calculating the person pose in the map plane, which is parallel to the floor. The pose has two components: position and orientation. The position of a person is easily extracted by selecting the XY components of the 3D point corresponding to the person central point. In the case of orientation, some extra steps are required. The XY components of the shoulders are selected and are joined by a line segment. The aim is extracting the orientation of the normal vector from the segment, which coincides with the person orientation, for which trigonometry is applied (see Figure 11a). A special case occurs when a person is placed sideways to the camera. In this case, the 3D points extracted from the point cloud could coincide for both shoulders, given that one of the shoulders is hidden (see Figure 11b). If only the shoulders information was used, an estimation of the orientation could not be performed because a slight change in depth measurements causes considerable changes in orientation. That is why the third point, corresponding to the nose, is used in these cases. Its position with respect to the shoulders is checked on the RGB image, as shown in Figure 11c. A different predefined orientation value is assigned to orientation depending on whether the nose is to the left or to the right of the shoulders.

The outcome of this procedure is a set of people poses that will be used for personal space modeling. However, an extra step needs to be performed before. Given that the robotic system contains multiple sensors, people are not only captured by the RGBD camera, but also by the rest of the sensors. That is why distance data captured by the 2D LiDAR corresponding to people need to be removed. For that purpose, 2D data inside a region of interest centered at each people’s position are removed. In this way, people are only accounted for once, and their location is not additionally seen as an obstacle.

##### Modeling Personal Space

The representation of personal space for a person or a group of people is a subject that has been studied and debated for a long time. Depending on various factors, such as the number of people, social aspects (age, culture, gender) or the relationships that humans have with each other, different types of distances are stipulated in which we humans communicate [33,34]. In general, four different types can be established, which are divided according to the user comfort level. These types are as follows:

- *Public distance:* This distance is defined for values over 210 cm. In this distance the communication needs to be with high voice volume and eye contact is minimized.
- *Social distance:* It is maintained during more formal interactions. Its value is between 122 and 210 cm and prevents all kinds of contact, except visual and auditory.
- *Personal distance:* This distance is maintained during interactions with people with a higher level of confidence than in the other two cases, for example, with friends. The value of this distance is 46–122 cm, generating a better capacity to interact, without any opposition.
- *Intimate distance:* The value of this distance is 0–46 cm. It is commonly used in close relationships, given that a clear invasion of the personal space of the other person occurs. Due to proximity, the vision is blurred and other sensory signals are used, such as touch.

For this project, setting distance limits are essential for two main tasks: differentiating individuals from groups and modeling their personal space mathematically during robot navigation. These values need to assure an equilibrium between avoiding bothering people and engaging them during interaction.

It was decided to set the minimum social distance as the group limit. This choice was established on the basis of different factors. One of them is the attempt to generalize the proposed method for all types of cultures that the robot may encounter. There is a clear difference between cultures [35], with Mediterranean cultures being more prone to relationships with smaller interpersonal distances, and Northern European or Asian cultures with larger interpersonal distances. Therefore, a number of people are considered to belong to the same group as long as there is a distance of at most 122 cm between two of the members. In the case of a person, the selected distance limit to model personal space is the minimum personal distance, setting 46 cm as the closest allowed distance during the human–robot interaction.

In order to work with these values, it is necessary to make a series of mathematical statements. First of all, it is considered that the whole navigation process will take place in a two-dimensional environment, represented by the Euclidean space, where a set of *N* people can be found, represented as $H=\left[H_{i}\;\ldots \;H_{N}\right]$. The state of a person *i* is composed by its position, heading and velocity $H_{i}=\left[x{{}^{i}}_{H},y{{}^{i}}_{H},\theta {{}^{i}}_{H},v{{}^{i}}_{H}\right]$.

**Figure 12:**
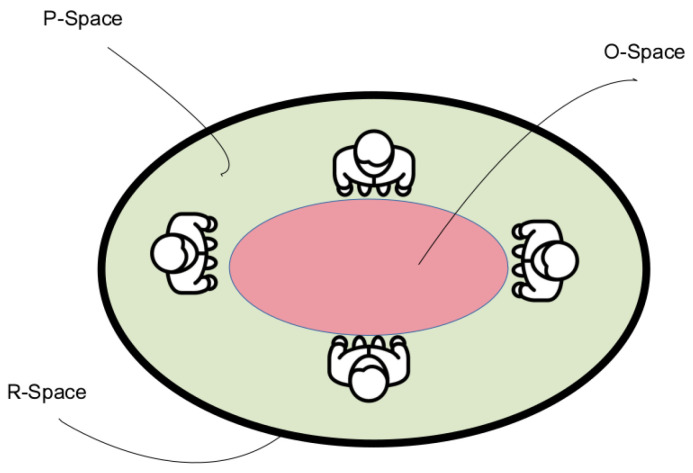
Representation of the different parts of the space for a group of people.

As previously described, two cases can occur: individuals and groups. Individuals are named $H_{ind}$, and their personal space is represented by $\Phi_{i}$. A group of people is represented by $H_{group}$. In this case, as the group shares a common space, they are modeled together. The O-Space represents the center space of the people that belong to a group. The P-Space represent the outer space that surrounds the O-Space and contains the people that belong to a group. The R-Space is the rest of space. This representation is visually explained in Figure 12.

After naming every element, the mathematical formulations for modeling each of the two cases are described below.

##### One Person Case

**Figure 13:**
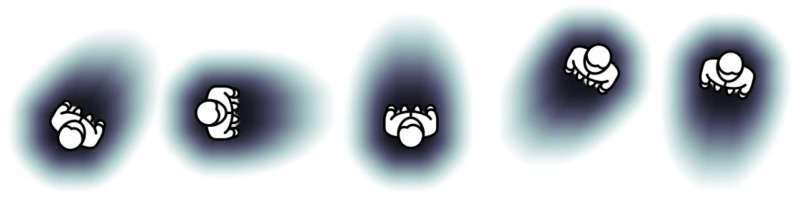
Representation example with two-Gaussian mixture model. The person is generated with different orientations in space.

For the representation of one individual, the two-Gaussian mixture model was used, given that it is one of the most used techniques in the literature [36]. The main idea of this method is to define the personal space that surrounds a person *i* with the mixture of two Gaussian functions, one of them to define the front of the individual $\Phi {{}^{F}}_{i}$ and the other to define the rear of the individual $\Phi {{}^{R}}_{i}$. The mathematical expression for a Gaussian function Φ is defined as follows:

(1) $$\Phi \left(q\right)=e^{\left(-\frac{1}{2}{\left(q-p\right)}^{\tau }\sum^{-1}\left(q-p\right)\right)}$$

where *p* defines the center of the Gaussian, representing the human position $p=\left(x{{}^{i}}_{H},y{{}^{i}}_{H}\right)$, ∑ represents the covariance matrix and *q* is every point that surrounds a human. The personal space using Equation (1) is defined as follows:

(2) $$\Phi_{i}\left(q\right)=\delta \left(y_{q}\right)\Phi_{i}^{F}\left(q\right)+\left(1-\delta \left(y_{q}\right)\right)\Phi_{i}^{R}\left(q\right)$$

where $q={\left(x_{q},y_{q}\right)}^{T}$ and $\delta (y_{q})=1$ if $y_{q}\geq 1$, which corresponds to the front of the person, and is 0 otherwise. For the Gaussian model, the covariance matrices are described as follows:

(3) $$\sum_{i}^{F}=\left(\begin{array}{cc} \sigma_{x}^{2} & 0\\ 0 & 4\sigma_{x}^{2} \end{array}\right)\;\;\;\;\;\;\;\;\;\sum_{i}^{R}=\left(\begin{array}{cc} \sigma_{x}^{2} & 0\\ 0 & \sigma_{x}^{2} \end{array}\right)$$

where the value of $\sigma_{x}^{2}$ is set using the individual distance limit, which is the minimum personal distance. Therefore, to model each of the axes, it is established that $\sigma_{x}^{2}=0.46/2=0.23$ m. In this way, a Gaussian is obtained in which the person is defined to a greater extent by the frontal part due to the fact that it is considered that the frontal personal space has a greater weight in human interaction since it is through it that humans can manipulate the environment, move around and perceive the surroundings. Figure 13 shows the representation of one person using the described model.

##### Group of People Case

**Figure 14:**
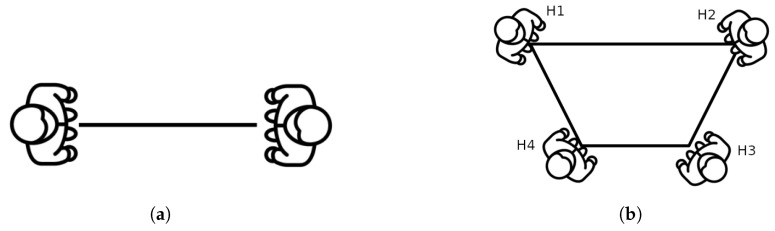
Examples of geometric entities for the description of a group of people. (**a**) For two people, the only geometric entity is a line; (**b**) For multiple people, the geometric entity will depend on their position.

As it was mentioned before, people closer than the specified distance limit are treated as a group. Depending on the number of people, groups will be modeled differently. Initially, groups are described by their simplest flat geometric structure. This means that each person is identified with a vertex, and they are joined such that the polygon edges do not intersect with each other. Using this definition, we call neighbors those people who are directly related with an edge. Therefore, the method is generalizable for any number of people. Figure 14 shows a visual example for the generation of the geometric entities. In the case of Figure 14b, H2 and H4 are neighbors of H1, but H3 is not because they are not directly related.

For the generation of the final model, the position of each person, represented by $H_{i}=\left(x_{i},y_{i}\right)$, is required. In contrast to previous methods, the orientation of a person in a group is not taken into account. It is decided because, after several comparisons, results obtained with and without taking orientation into consideration generate almost identical results. Therefore, it is assumed that it is not a factor whose relevance should be considered, so a person belongs to a group only if the distance requirement is met. This distance, denoted as $D_{v}$, is calculated using the Euclidean distance between the neighbors of the person and has to fulfill $D_{v}\leq 1.22$ m. That restriction must be complied with by at least one of the neighbors of each person of the group. For the correct O-Space representation, it is decided to additionally consider an estimation of the mean distance between each neighbor, $D_{H}$.

Another necessary element is the center *C* of the group. In previous works, it was represented as the point where all the bodies are pointing. However, in our method, the center is generalized as the centroid of the polygonal shape, assuming that every component of the group is looking in that direction. In the case in which the group is only formed by two people, the middle point of the line joining them is selected.

Finally, distance $D_{i}$ is defined as the Euclidean distance corresponding to the person of the group that is further away from the center, which is represented as $H^{far}$.

Knowing the previous values, the O-Space can be modeled using a two-dimensional Gaussian $\Phi_{i}$ as follows:

(4) $$\Phi_{i}\left(Q\right)=e^{\left(-\frac{1}{2}{\left(Q-C\right)}^{\tau }\sum^{-1}\left(Q-C\right)\right)}$$

where *C* is the center of the Gaussian and *Q* represents the point which is being evaluated. The covariance matrix ∑ is defined as

(5) $$\sum =\left(\begin{array}{cc} \sigma_{x}^{2} & 0\\ 0 & \sigma_{y}^{2} \end{array}\right)$$

with $\sigma_{x}=D_{H}/4$ and $\sigma_{y}=D_{i}/2$. In order to obtain the value of $D_{H}$, the mean distance between neighbors is used, as long as they meet the maximum distance condition. The equation that describes this value is

(6) $$D_{H}=\frac{1}{N}\left(\sum_{i,j=1}^{N}\left(D_{v_{i,j}}\right)\right)$$

where $D_{v}$ represents each of the sides of the polygon that are created for the group (distance between each pair of neighbors), and *N* represents the total number of sides of the polygon.

The equation that defines $D_{i}$ is

(7) $$D_{i}=\sqrt{{\left(H_{x}^{far}-C_{x}\right)}^{2}+{\left(H_{y}^{far}-C_{y}\right)}^{2}}$$

where $H^{far}$ represents the further person of the group. This person is considered as the pivot of the Gaussian model, generating the angle Θ that is needed to rotate to have the correct model orientation. Θ is calculated using the following equation:

(8) $$\Theta =\arctan\frac{H_{x}^{far}-C_{x}}{H_{y}^{far}-C_{y}}$$

**Figure 15:**
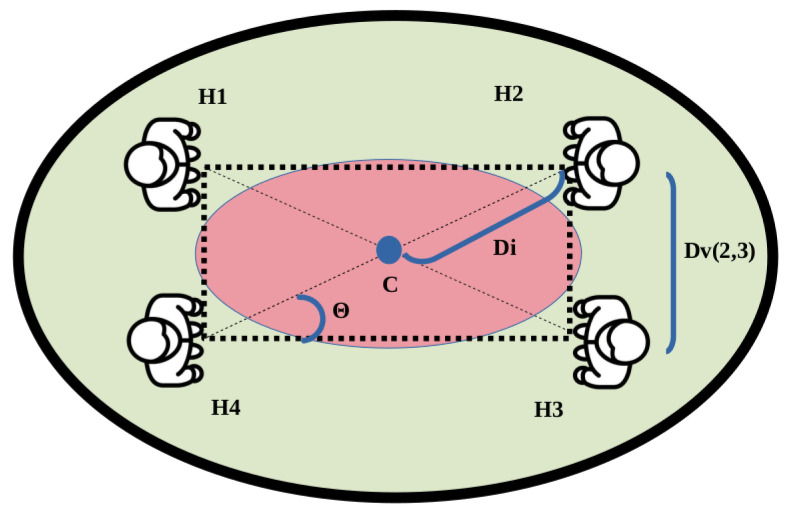
Example of polygonal representation. To simplify the explanation, a regular polygon is described. The same color scheme as in Figure 12 has been applied.

This value allows to correctly orientate the Gaussian model. The idea of using $H^{far}$ and Θ allows the method to accommodate any person within the Gaussian model regardless of their position. This is especially relevant when the method generates an irregular polygon, allowing to delimit all persons under the same Gaussian. A schema of the proposed model is described in Figure 15.

**Figure 16:**
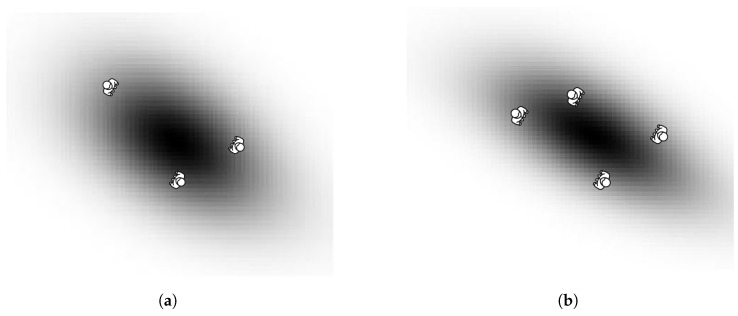
The method group together the different humans under the same Gaussian model. (**a**) Example of a Gaussian generated for a group of 3 people; (**b**) example of a Gaussian generated for a group of 4 people.

An example of the method applied for groups of three people and four people is shown in Figure 16. It can be noticed that the Gaussian models are rotated according to the people location, so all group members are included.

##### Inclusion of the Gaussian Models to the Velocity Map

**Figure 17:**
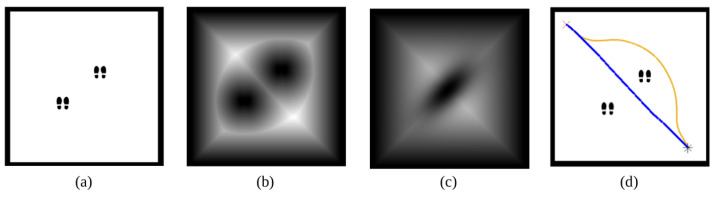
Advantages of using the proposed Gaussian model method. For the environment presented in (**a**), the velocity map *F* is extracted, interpreting humans as objects (**b**) and as a group (**c**). When the path planner is applied (**d**), the calculated path goes between the two individuals in the first case (blue line), whereas for the second case, the social space is respected (orange line).

The next step to obtain a social-aware path is adding the previously explained Gaussian model into the velocity map derived from the FM2 planner. The novelty of the method is the idea of distinguishing between the addition of an object to the occupancy grid map and the addition of a person or a group of people to the velocity map. Normally, when an obstacle is detected by any sensor during robot navigation, it is included in the occupancy grid map as an object, directly adding its shape, so the local planner can reconfigure the planned path. By contrast, our method allows to add the person or the group directly in the velocity map (*F*) in the shape of their personal space. If instead of adding the person or the group to the velocity map, it was added to the occupancy grid map, each person would be treated individually as an object, generating a modification on the velocity map derived from their individual shapes that does not assure a social-aware path. An example of that is modeled in Figure 17, where the path calculated by treating people as objects (blue line) goes through a group, interfering unnecessarily in their social interaction. With our method, the path surrounds the group (orange line).

For the addition of the Gaussian model to the velocity map, it is necessary to transform from the robots frame to the global reference. For that purpose, its necessary to know the $R=\left[x_{R},y_{R},\theta_{R}\right]$ global reference frame. Once that the pose of the Gaussian model in the global frame is known, the addition of this model is performed using an image mask. That image mask is defined as the local model of the Gaussian. The equation for that process is the Haddamard product [37]:

(9) $${\left(A⊙B\right)}_{i,j}$$

where *A* and *B* are the global map and image mask, respectively, and i,j represent each of the elements for each matrix. For that process, two other requirements are necessary. First of all, it is necessary to re-scale the local map. For that, the resolution of the global map is known, and the resolution of the mask applied is selected as a standard value for all the possible cases.

Second of all, for the Haddamard product, both matrices have to be the same size. Normally, the global map is bigger than the local matrix or mask applied. A local frame of the global map is selected with the same size that the mask applied. To determine the exact part of the global map where the mask needs to be applied, is necessary to know the orientation of the robot. Knowing that value, with respect to the global frame, the method is able to orientate the Gaussian model correctly with respect to the robot.

## 3. Results

**Figure 18:**
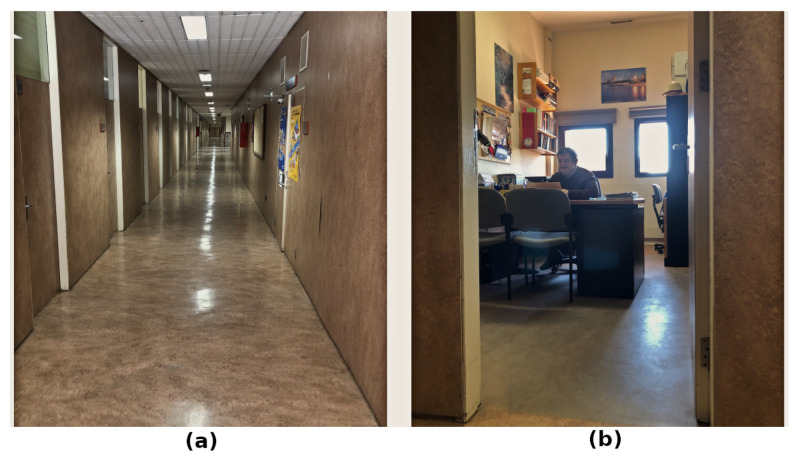
Real scenario for testing the proposed method: (**a**) corridor, (**b**) office.

The aim of this section is validating the proposed method on a real scenario. The selected scenario is a working zone with a long corridor and several offices, as shown in Figure 18. This zone has several challenging characteristics. The floor has no roughness, which could cause wheel drifts, and a high number of rotations are required to enter rooms, which causes cumulative errors in odometry. Additionally, the corridor is almost symmetric, and offices are cluttered with multiple furniture pieces, which may cause issues when localizing the robot and executing path-following techniques. Finally, multiple dynamic elements, mostly people, are expected.

Initially, the robot was teleoperated through the scenario, where only some of the office doors were open. Three-dimensional data were captured to map the environment. Once the map system is available, three experiments are performed: the navigation strategy without the social module is tested, people detection and modeling is performed while the robot is standing still, and finally everything is integrated in the navigation system to consider people dynamically, proving that their personal space is preserved.

### 3.1. Mapping an Indoor Scenario

**Figure 19:**
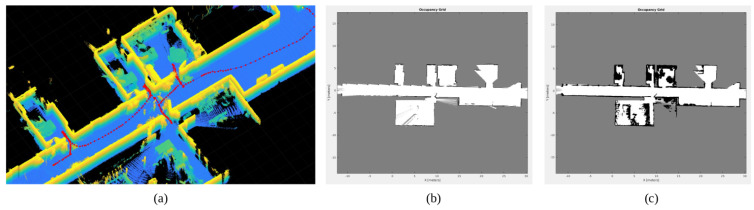
Mapping results: (**a**) global point cloud, where red dots are corrected robot poses, (**b**) layout grid map, and (**c**) navigation grid map.

The first step for performing experiments is to map the scenario in which the robot will be moving. As mentioned before, the robot was teleoperated around the scenario while capturing data from odometry and the 3D LiDAR. Results from applying SLAM based on Harmony Search are presented in Figure 19a, where the ceiling is removed to better appreciate the results. Thanks to this algorithm, robot poses are corrected, and scans are aligned. The result is a global point cloud with benefits, such as thin walls, well-defined doors and aligned rooms. Multiple objects can also be observed in the figure, where blue colors correspond to lower zones and yellow ones are data captured at higher heights. The second stage on the mapping procedure is extracting 2D occupancy grid maps from the global point cloud. The selected slice for defining the layout of the zone is between the robot’s height and the ceiling. The resulting map is shown in Figure 19b. Rooms are clearly defined, and noise is highly reduced with respect to traditional occupancy grid maps, which helps with identifying narrow passages. The second data slice defines the navigation space for the robot. In this case, data are selected from the floor to the robot’s height. The resulting map, illustrated in Figure 19c, includes all objects in the specified slice. This is a relevant aspect since multiple offices are found in the selected scenario, where a high number of tables and chairs are found. Hence, not only legs are included in the map, benefiting the following applied navigation techniques.

**Figure 20:**
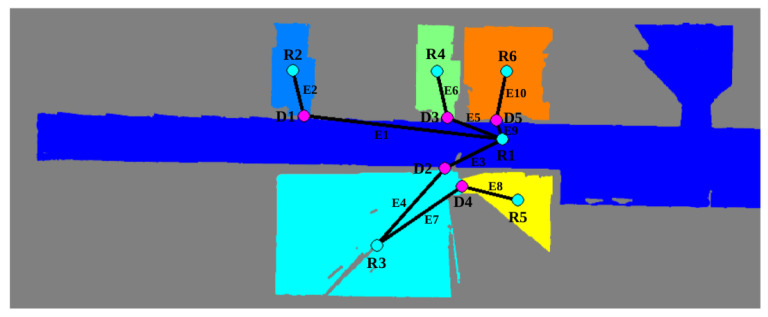
Resulting topological map overlapped with the segmented scenario. R and D correspond to room and door nodes, respectively, and E corresponds to edges.

The last step is extracting the topological map from the layout occupancy grid map to represent the location of rooms and doors. Results are provided in Figure 20, where the resulting topological graph is overlapped with the labeled map and annotated for a better understanding. Labeled nodes starting with an R correspond to geometrically segmented rooms, whereas nodes starting with a D correspond to door nodes. It is worth recalling that every door is comprised of a set of two symmetrically placed nodes, but for representation purposes, they are merged into a single one. Finally, edges are marked with letter E and clearly indicate connectivity among regions.

### 3.2. Single-Sensor Navigation Strategy

In this section, the proposed navigation system based on the geometric and topological maps is launched on the robot to move around the indoor scenario. In this case, only the 2D LiDAR is used to detect discrepancies between the map and real-time detected objects. This is useful for both static and dynamic objects. It will be also used for door trespassing when required.

**Figure 21:**
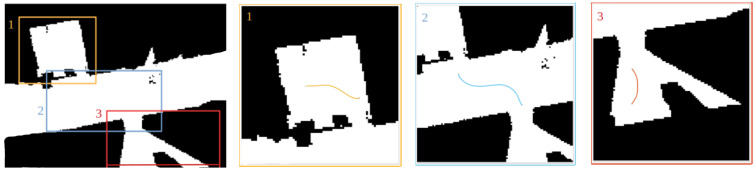
Calculated local geometric paths. The robot is located in room 1 and it needs to reach room 3, so a path in room 2 is also computed to connect both locations.

The robot is initially placed in one of the office rooms and is commanded to go to another one in the opposite side of the corridor. Hence, the topological planner indicates that the robot needs to geometrically navigate in three different rooms (two offices and one corridor), connected by two doors. Results of the local geometric plans can be observed in Figure 21. It can be noticed that the paths do not reach the center of the narrow zones. This space will be later traversed using the corresponding topological nodes with the door trespassing strategy.

**Figure 22:**
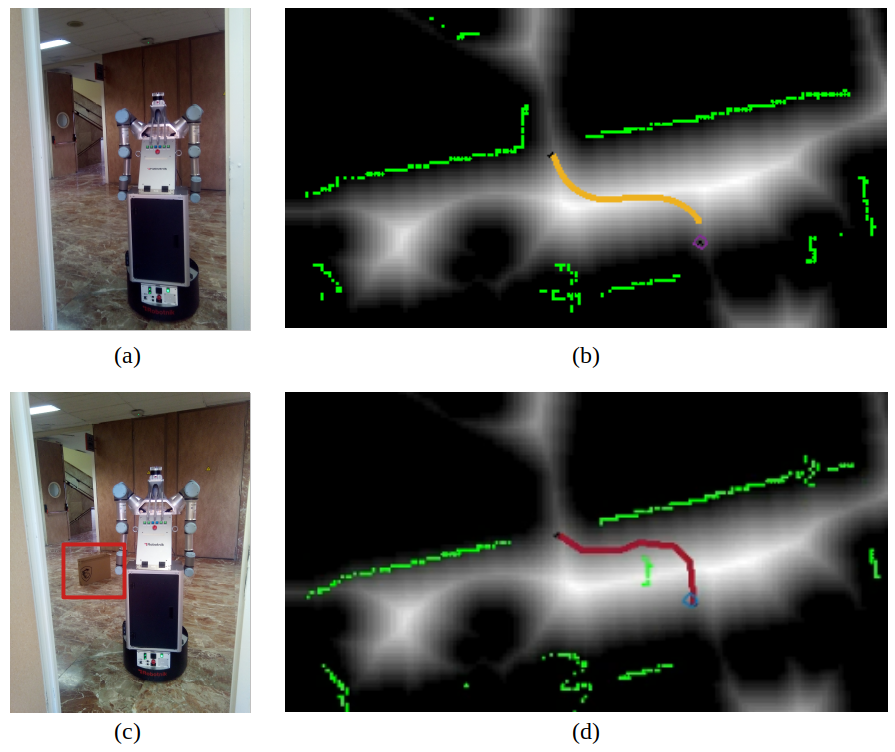
Replanning behavior: (**a**) initial scenario, (**b**) initial planned path (yellow) on the velocity map, where green is 2D LiDAR data, (**c**) modified scenario with an unmapped box, (**d**) modified path considering the box.

Room number 2, corresponding to a corridor, is selected to test the dynamic behavior of the navigation system. While the robot is moving, a box is placed in the middle of the planned path, as shown in Figure 22, where (a) and (b) correspond to the initial test scenario and (c) and (d) show the box location and the replanned path. The 2D LiDAR, marked in green, successfully detects the new object and replans to avoid it.

**Figure 23:**
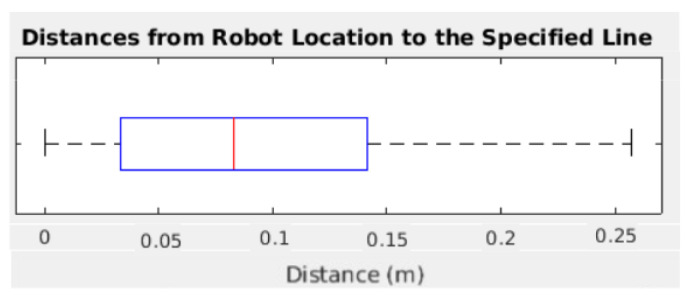
Box plot representing the deviation distance in meters of the robot with respect to the desired path joining the two door nodes.

Finally, the door-crossing behavior is tested along several runs. The robot is commanded to move from one side of the door to the other, and its geometric location is saved. The distance between the commanded path and the executed one is calculated and analyzed as shown in Figure 23. The robot successfully traverses doors without colliding, with a deviation from the original plan of less than 0.15 m in 75% of cases. Bigger values correspond to the robot approaching the planned path, so it is not close to the door yet and the collision risk is minimal. A comparison between this method and purely using FM2 is not possible. When trying to cross a door with the geometric planner, the majority of the times, the robot hit the walls near the door because it does not go through the exact door center.

### 3.3. People Detection and Modeling Performance

**Figure 24:**
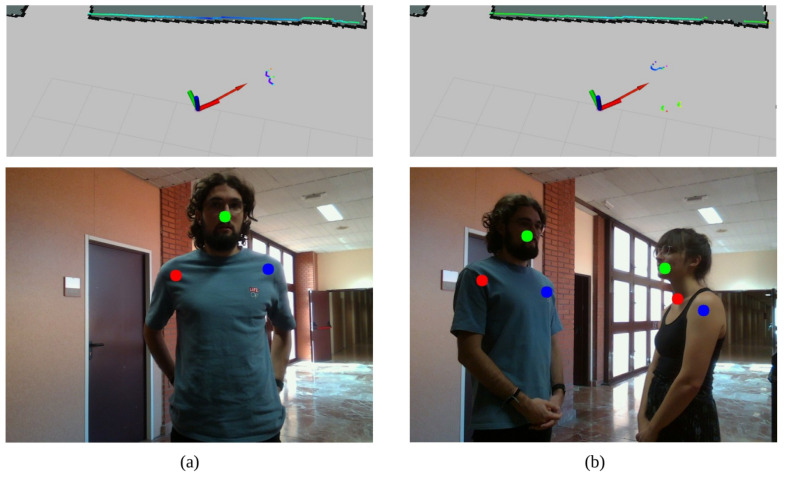
People detection: (**a**) a person approaches the robot, so its feet are detected by the 2D LiDAR (**top**, where the red arrow is the robot position and green/blue zones are 2D LiDAR data) and its shoulders and nose are detected by the RGBD camera (**bottom**, where the red dot is the right shoulder, green is the nose and blue is the left shoulder), (**b**) a group is formed, so two sets of feet (**top**) and two torsos (**bottom**) are detected.

The third performed experiment consists on checking the performance of the people detection and modeling module. In this case, only the RGBD camera is used. The robot is standing still while checking for people around to include it in the velocity map *F*. Two situations are selected for checking how people are considered: a person and a group of people talking in front of the robot. This is depicted in Figure 24, where both the 2D LiDAR (top images) and the RGBD camera (bottom images) data are provided for ensuring that they are aligned. The robot pose is represented by the red arrow in the map, whereas greenish dots correspond to LiDAR measurements. Regarding bottom images, they correspond to captured data from the RGBD camera, where green dots represent noses, red dots indicate where the right shoulder is placed, and the left shoulder is marked with a blue dot. This is invariant to the person orientation, so left and right shoulders are always differentiated.

**Figure 25:**
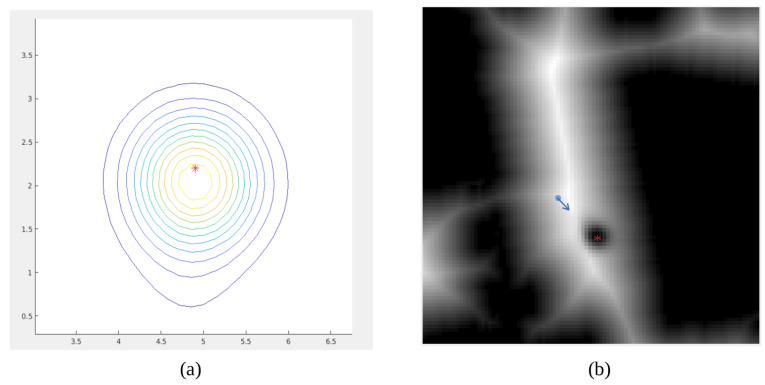
Detection and modeling of a person: (**a**) Gaussian model, in meters, defined in the robot reference frame, (**b**) inclusion of the Gaussian model in the velocity map using the map reference frame.

Results corresponding to the first case are shown in Figure 25. The person is first detected and its 3D pose is estimated using the camera point cloud. Then, it is locally modeled using Gaussian mixture model considering the robot base as the reference frame. This is depicted in Figure 25a, where the red star is the center of the Gaussian. It can be observed that the person orientation is considered and the Gaussian is oriented with its bigger side corresponding to the front part of the person. On a second stage, the Gaussian model is included in the velocity map considering the robot pose. This is shown in Figure 25b, where the blue arrow indicates where the robot is located and the red star marks the center of the Gaussian. Velocity around the red star is reduced using the Gaussian shape, ensuring that the person space will not be traversed during navigation.

**Figure 26:**
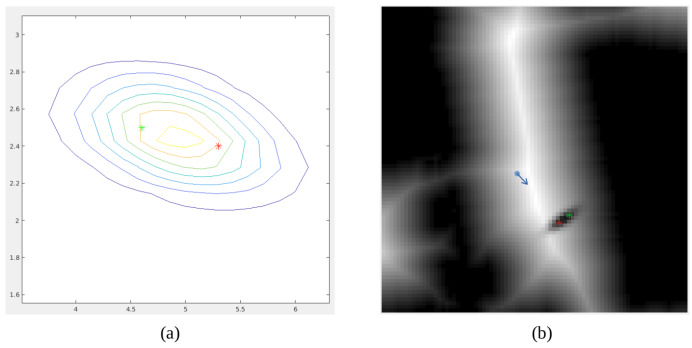
Detection and modeling of a group: (**a**) Gaussian model, in meters, defined in the robot reference frame, (**b**) inclusion of the Gaussian model in the velocity map using the map reference frame.

In the second case, two people are detected, so the Gaussian model varies. In this case, no information about people orientation is required. However, the Gaussian model is oriented according to the location of each of the participants in the group. Results are shown in Figure 26a, where the green and red stars indicate where people are detected. Given that the person on the left is slightly further away from the robot, the Gaussian is rotated so that the two detected people are symmetrically placed inside it. This model is included in the velocity map as shown in Figure 26b. It can be noticed that the central part between both people corresponds to almost zero velocity. This means that the robot will always respect the space between them, so the calculated paths will only go around the group.

### 3.4. Multi-Sensor-Based Social Navigation

Once the navigation strategy and the people detection module performance have been tested, the final experiment merges all sources of sensor information for social-aware robot navigation. The following shows how the robot performs when dealing with multiple dynamic events. Additionally, results can be seen in the following link: https://youtu.be/qCg3jC__fO4 (accessed on 10 October 2022).

**Figure 27:**
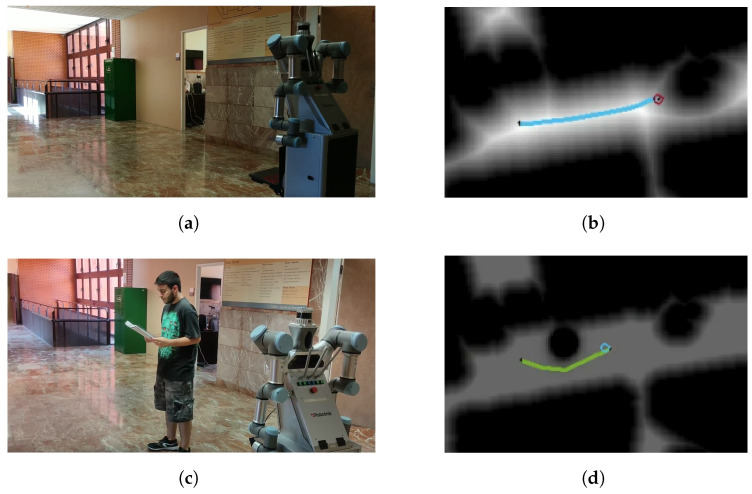
Social navigation example of the robot in a real scenario with a person. (**a**) Initial scenarios without people; (**b**) initial planned path; (**c**) person detection in front of the robot, standing in the initial path; (**d**) path replanning considering the personal space of the person.

Initially, it is tested with one person, as shown in Figure 27. The scenario is first empty, with no objects or people around (Figure 27a). FM2 calculates the initial path that the robot has to follow to reach the specified goal (Figure 27b, where the circle is the robot location). During the plan execution, a person is placed in front of the robot, interfering with the path (Figure 27c). The RGBD camera detects the person, and their social space is included in the velocity map, modifying the initial path (Figure 27d). The velocity map is additionally saturated to obtain a smoother path. The robot is capable of avoiding the person without being intrusive.

**Figure 28:**
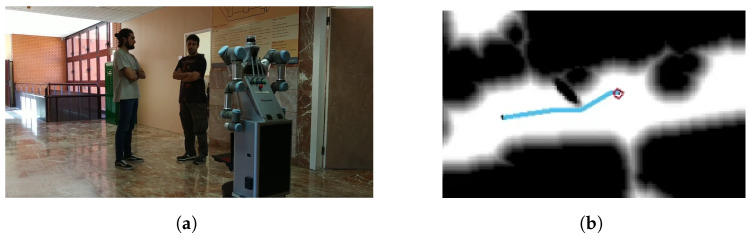
Social navigation example of the robot in a real scenario with two people. (**a**) Initial scenario with two people standing in the initial path; (**b**) path replanning considering the interaction space.

The same experiment is performed with a group of two people. The robot has to follow the same initial path as before but in this case the social space is modeled differently. Results are shown in Figure 28. People in the scene are close enough to be considered a group but far enough to leave space so that the robot fits between them (Figure 28a). Using the proposed Gaussian modeling strategy, the velocity matrix is zero between them, so planning in that region is prohibited. The replanning procedure successfully surrounds the group without interrupting their interaction (Figure 27b).

**Figure 29:**
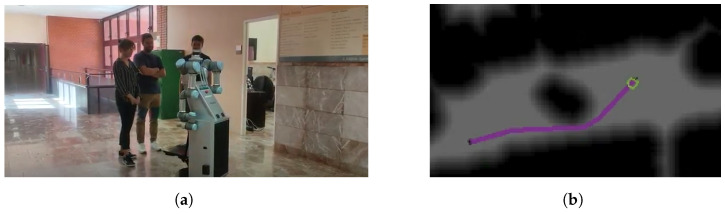
Social navigation example of the robot in a real scenario with three people. (**a**) Initial scenario with a group of three people interfering with the initial path; (**b**) replanning of the path acoording to their personal space.

The case in which a group with more than two people is detected is shown in Figure 29. As it happened before, the group is standing in the center of the space, interfering with the initial plan (Figure 29a). Their social space is modeled, and the path is replanned to avoid them without disturbing their conversation (Figure 29b). It can be seen that the resulting Gaussian is bigger than in the previously performed experiments, so the robot has to get very close to the corridor wall. Even so, the robot successfully avoids them and achieves its target.

**Figure 30:**
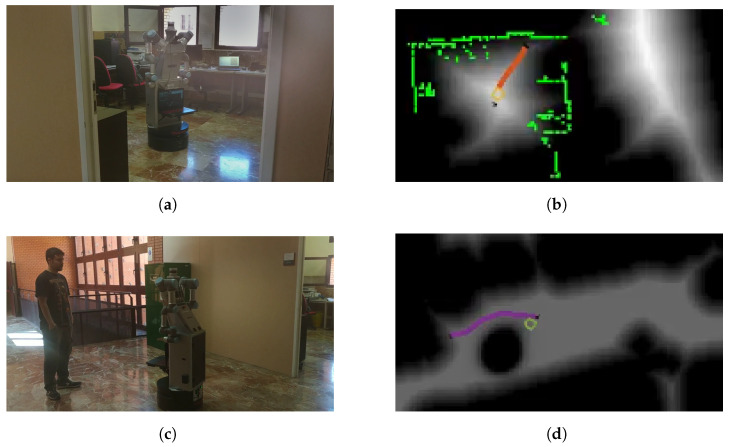
Final experiment with door trespassing and person detection. (**a**) Robot navigating to exit the room; (**b**) path (red) to move to the coordinate corresponding to the door topological node (green corresponds to LiDAR measurements); (**c**) detection of a person in front of the robot that stands in the path.; (**d**) path replanning considering the personal space of the detected person.

As a final experiment, it is intended to test how fast the social navigation strategy works once the robot enters a new room. The robot is located in one of the offices and is commanded to move to a specific coordinate in the corridor. Hence, two local geometric paths are calculated, one to exit the office and another one, in the corridor, to reach the final point. The robot starts inside the office (Figure 30a). No people are found at that time in the place, so the robot directly follows the initial path (Figure 30b). Right after concluding the subsequent door crossing behavior, a person is detected in the corridor path (Figure 30c). Its detection could not have been previously done because the person was occluded by walls. However, once the robot is outside, it immediately detects the person and finds a path that respects social distancing (Figure 30d). One of the main advantages of using FM2 and Gaussians is that the path is smooth and the robot is not trapped in any local minima, so the robot is not required to move backwards or sideways. This is why the omnidirectional base is well-suited for challenging situations such as the one presented in this experiment. In the case in which the robot is trapped, it can turn on the spot until the path is clear and it can move forward.

## 4. Discussion and Conclusions

In this work, a method for socially navigating a scenario using multiple sensors is presented. This method is divided into four modules: mapping, localization, path planning and plan execution. Their results are reported in Section 3.

Mapping results are provided in Section 3.1. It is proven that using a 3D LiDAR overcomes results from using a single 2D LiDAR for mapping. The versatility of using 3D data allowed us to extract multiple occupancy grid maps at different heights, as shown in Figure 20. While data at a higher height create a map with no furniture, using data at a lower height allows to consider the whole objects structures. Finally, in this module a topological map is extracted by benefiting from the absence of noise from the higher occupancy grid map, clearly locating doors.

In Section 3.2, the complete navigation strategy solely based on the 2D LiDAR for the dynamic behavior is tested. The robot is capable of locally planning on each separate room and avoiding obstacles when required, as depicted in Figure 22. In the example case, the unexpected object is a box, which produces a modification of the orange path into the red one. Even though the robot does not collide with the box, it can be observed that the red path is very close to it. In the case in which it was a person, he or she could have felt uncomfortable. Considering door trespassing, it is proven that the proposed strategy improves the navigation system by avoiding collisions in narrow zones.

Section 3.3 shows the results of detecting and modeling people and Section 3.4 coordinates this with the navigation system. It is proven that the replanning strategy correctly modifies paths to not only avoid people, but also to respect the specified social distance while providing continuous and smooth paths. This is tested with one and more than one person, as well as in challenging situations, such as detecting a person right after trespassing a door. This is a major improvement with respect to state-of-the-art techniques, which tend to discretize both the Gaussian model and the path. Other works also address the problem of fusing data from sensors, such as 2D laser scanners or RGBD cameras, to socially navigate. The work in [38] proposed data fusion from a fish-eye camera, a RGBD camera and a 2D laser scanner for people modeling and tracking. Additionally, face descriptors are extracted to re-identify users. Authors in [39] presented a real-time collision avoidance system in which the fusion of a RGBD camera and a 2D LiDAR is performed. The main purpose of that sensor fusion is to obtain the model of objects or people to be avoided and, using the 2D LiDAR, to move through them using empty spaces. In contrast, our method goes beyond solely modeling people or avoiding obstacles, and it achieves social convention compliance, in particular, respecting personal and social interaction space.

Overall, it can be stated that navigation strategies highly benefit from using synchronized muti-sensor systems. Additionally, modeling people using the Gaussian mixture model and considering its continuous values in the fast marching velocity map results in smooth paths, which can be dynamically replanned.

As future work, it is proposed to include a person tracker to avoid duplicating people while dynamically detecting them and predicting the next person’s location. In the cases in which a person moved too fast in front of the robot, it was sometimes considered to be two individuals or a group of two people. Moreover, the algorithm performance could be improved by translating it from MATLAB to a faster language, such as Python or C++.

## Data Availability

The data presented in this study are available on request from the corresponding author. The data are not publicly available due to privacy restrictions.
